# Electronic chirality inversion of lanthanide complex induced by achiral molecules

**DOI:** 10.1038/s41598-018-34790-0

**Published:** 2018-11-06

**Authors:** Satoshi Wada, Yuichi Kitagawa, Takayuki Nakanishi, Masayuki Gon, Kazuo Tanaka, Koji Fushimi, Yoshiki Chujo, Yasuchika Hasegawa

**Affiliations:** 1 Graduate School of Chemical Sciences and Engineering, Hokkaido University, N13 W8, Kita-ku, Sapporo, Hokkaido 060–8628 Japan; 2 Faculty of Engineering, Hokkaido University, N13 W8, Kita-ku, Sapporo, Hokkaido 060–8628 Japan; 3 Faculty of Industrial Science and Technology, Tokyo University of Science, 6-3-1 Niijuku, Katsushika-ku, Tokyo 125-8585 Japan; 4 Graduate School of Engineering, Kyoto University, Katsura, Nishikyo-ku, Kyoto, 615-8510 Japan

## Abstract

A novel mechanism for chiroptical activity inversion based on the electronic structure of metal complexes without Λ- or Δ-type structure change was demonstrated spectroscopically and theoretically. To demonstrate the mechanism, a europium (Eu(III)) complex with chiral (+)-3-(trifluoroacetyl)camphor (+tfc) and achiral triphenylphosphine oxide (tppo) was prepared. The steric and electronic structures of the Eu(III) complex were adjusted by additional achiral tppo and coordinating acetone molecules, and were characterised by ^1^H NMR, photoluminescence, and emission lifetime measurements. The optical activity of the Eu(III) complex in solution was evaluated by circularly polarized luminescence (CPL) measurements. CPL sign inversion, which was independent of Λ- or Δ-type structure changes from the spectroscopic viewpoint, and a drastic CPL intensity enhancement were observed depending on the external achiral molecules around Eu(III) ion. These phenomena provide the first clarification of optical activity change associated with electronic structure rather than chiral coordination structure-type (Λ or Δ) under external environments.

## Introduction

Chirality is prevalent in our life. Chirality is attributed to asymmetric molecular structure, which arises from the molecular level to the macroscopic helical scale, like a DNA double helix^1,2^. Chiral structures also play a critical role in chiral properties such as enzyme activity and asymmetric synthesis using chiral catalysts^3–5^. The chiral structures are susceptible to external environments such as solvents^6^, temperature^7^, light^8^, or pH^9^, causing chiral inversion phenomena in large variety of systems from biological macromolecules like B- or Z-form DNA^10^ to artificial soft matter and liquid crystals^11–14^. Hence, the influence of external environment is of importance to control the molecular chirality.

Generally, chiral molecules exhibit optical activities with absorption (circular dichroism: CD) or emission (circularly polarized luminescence: CPL) related to the molecular structure^15^. Optical activities are estimated from the different electronic transition probabilities between left- and right-handed circularly polarized light. The CD and CPL signals are dependent on the molecular chirality, which is influenced by the external field. The inversion of optical activity induced by the external field has been reported extensively^16–25^. Ishii and Verbiest achieved the CD signal inversion of a chiral polythiophene derivative by controlling the molecular aggregation speed^16^. George observed the tuneable helicity inversion of Zn(II)-coordinated naphthalenediimide assemblies with chiral adenosine phosphates using CD measurements^17^. Meskers and Swager described the solvent polarity-dependent CPL signal switching of a self-assembled chiral poly(*p*-phenylenevinylene) derivative^18^. The magnitude of optical activity is estimated by a dissymmetry factor (*g*), which is composed of electric and magnetic field components related to the electronic transition^15^. Their components are governed by the molecular structure in the surrounding environment.

**Figure 1:**
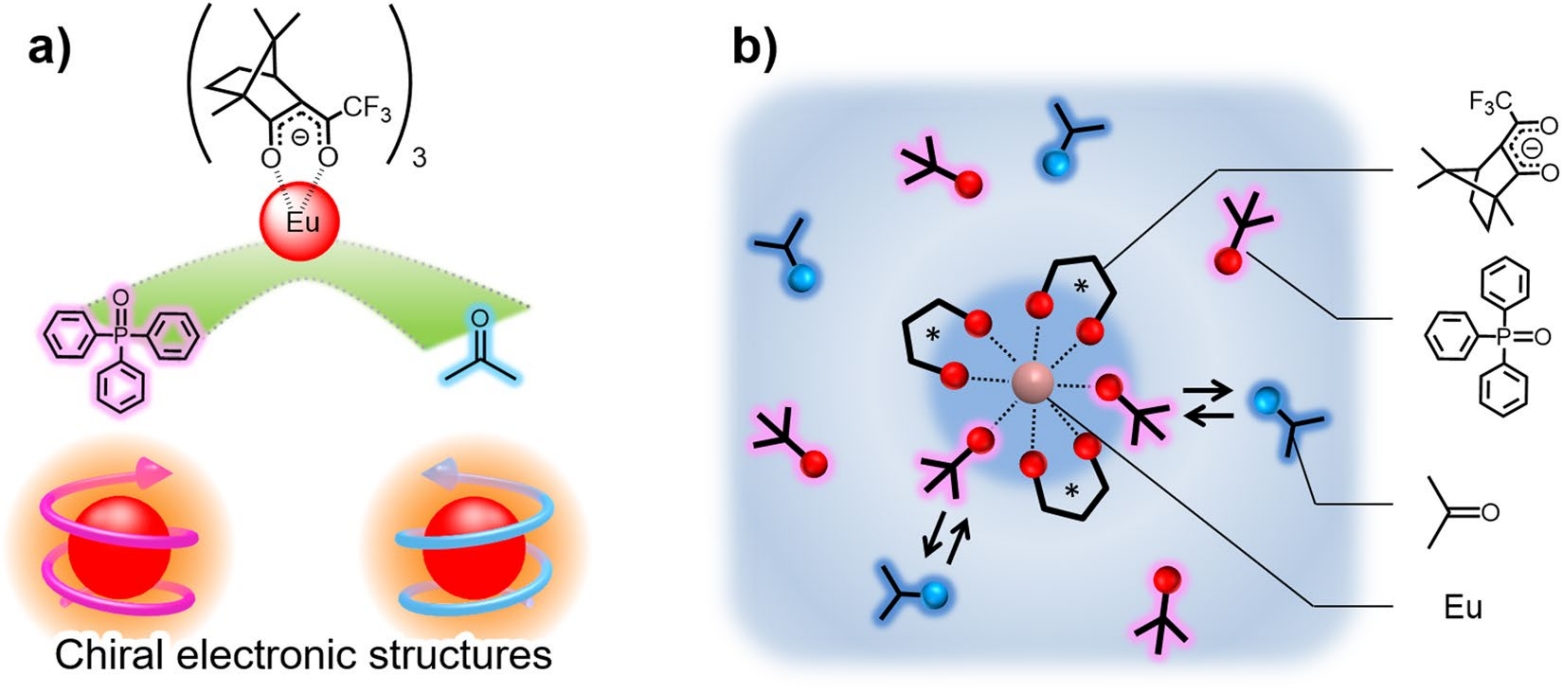
Schematic images of (**a**) chiral electronic structures depending on the external molecules around the Eu(III) ion and (**b**) the coordination structures of the Eu(III) complex depending on external achiral molecules around the Eu(III) ion in solution.

Here, we targeted chiral lanthanide complexes to clarify the influence of electric and magnetic field components on optical activities. Their sharp luminescence bands comprise electric and magnetic field components of 4f-4f transitions^26^. Their components in the electronic transitions are dominated by external organic molecules around the lanthanide ion. In particular, the magnetic dipole transition of the europium (Eu(III)) complex exhibits a large dissymmetry factor on the CPL (*g*_CPL_) by the introduction of chiral organic ligands; this is established by the effective contribution of the magnetic field component^27–30^. The optical activity signal of the lanthanide complex with chiral ligands has been evaluated by Λ- or Δ-type coordination structures previously^31,32^. Yuasa and Parker reported the chiroptical activity inversion of chiral Eu(III) complexes influenced by the different steric structures of achiral molecules^33,34^, which implied steric inversion between Λ- and Δ-type structures. Note that chiroptical activity is related to the electronic transition of the Eu(III) complex; therefore, the magnitude and sign could be also affected by the chiral electronic structure of the Eu(III) ion surrounded by external ligands (Fig. 1a).

In order to demonstrate the chiroptical activity based on the chiral electronic structure in external environments, we choose the chiral Eu(III) complex with bidentate (+)-3-(trifluoroacetyl)camphor (+tfc) and monodentate triphenylphosphine oxide (tppo) as the chiral and external achiral ligands, respectively. The Eu(III) complex [Eu(+tfc)_3_(tppo)_2_] (Δ-type structure, as determined by X-ray single-crystal analysis) shows a large *g*_CPL_ (−0.47) in acetone-d_6_^35^. The chiral electronic structure of the Eu(III) complex in solution was adjusted by additional achiral tppo and acetone molecules (Fig. 1b). The coordination and electronic structures in liquid media were characterised using ^1^H NMR, photoluminescence, and emission lifetime measurements. The chiroptical activities of the Eu(III) complex under external environments were evaluated using CPL measurements. In this study, we demonstrate a mechanism for the optical activity inversion based on the chiral electronic structure of the Eu(III) complex without Λ- or Δ-type structure change spectroscopically and theoretically, for the first time.

## Results

### Coordination structures in solution

**Figure 2:**
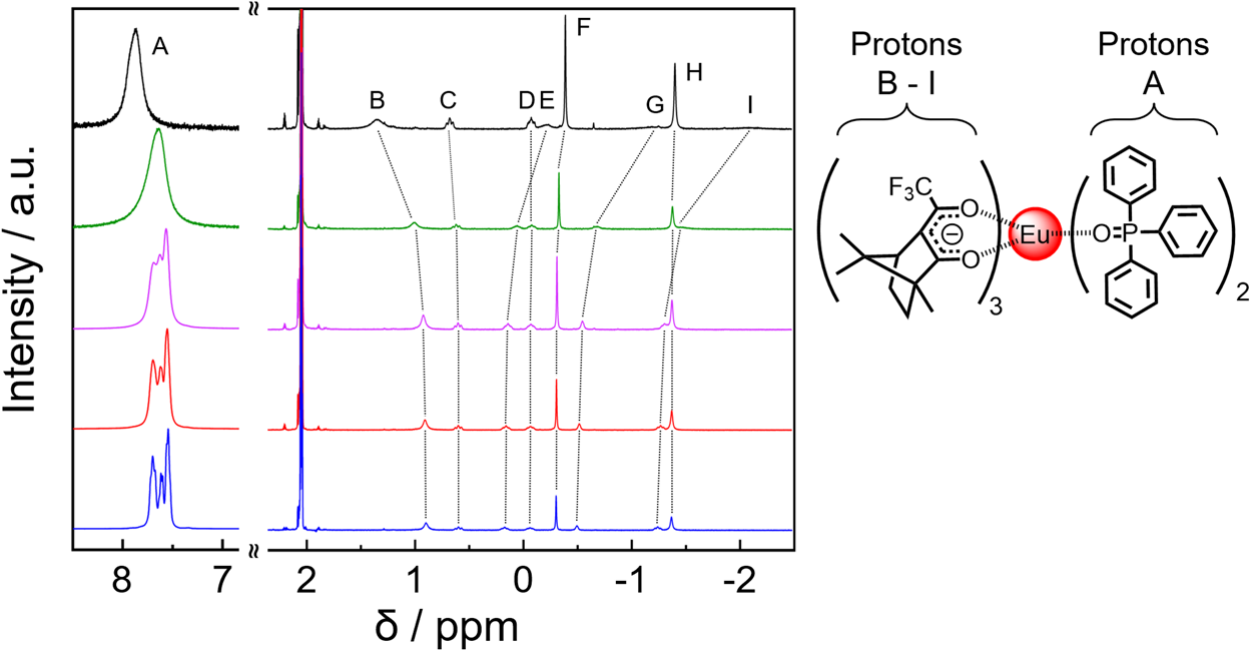
^1^H NMR spectra of **Eu**(+)-Ex0 (black), **Eu**(+)-Ex8 (green), **Eu**(+)-Ex28 (purple), **Eu**(+)-Ex48 (red), and **Eu**(+)-Ex98 (blue) in acetone-d_6_ (**Eu**(+); 1 × 10^−3^ M).

X-ray crystallography measurements indicated that the coordination structure of the Eu(III) complex in the solid is an eight-coordinated Δ-type structure composed of three chiral +tfc and two tppo ligands^35^. To evaluate the conformation of [Eu(+tfc)_3_(tppo)_2_] (**Eu**(+)) in acetone, ^1^H NMR spectra with additional *n* equivalents (*n* = 0, 8, 28, 48, and 98 relative to **Eu**(+)) of tppo molecules, namely **Eu**(+)-Ex*n*, were acquired (Fig. 2 and Supplementary Table S1). In the low-magnetic-field side, protons of tppo molecules in **Eu**(+)-Ex0 show broad peaks in Fig. 2 (black; A). The line-broadening and chemical shifts originate from the exchange reaction and paramagnetic effect on the metal complex^36,37^. The paramagnetic effect of the Eu(III) ion generally induces little broadening (bandwidth; nearly 10 Hz)^37^. Therefore, the large broadening of tppo signals in our experiment (Fig. 2, black; A, bandwidth; nearly 300 Hz) is mainly caused by their exchange reaction in acetone-d_6_. The lower-magnetic-field shift in **Eu**(+)-Ex0 (Fig. 2, black; A) is influenced by the direct coordination of tppo ligands with the Eu(III) ion.

NMR peaks of the chiral +tfc ligands in the Eu(III) complex were observed in the high-magnetic-field side (Fig. 2, signals B - I). **Eu**(+)-Ex8 (green) provided effective chemical shifts of +tfc ligands compared with those of **Eu**(+)-Ex0 (black). We also observed gradual shifts at the B, E, G, and I peaks of **Eu**(+)-Ex28 (purple), -Ex48 (red), and -Ex98 (blue). The effective shifts of the tppo and +tfc signals indicate that the Eu(III) complex with tppo molecules is rearranged by additional tppo molecules, resulting in the formation of several equilibrium states in acetone-d_6_.

### Luminescence properties

**Figure 3:**
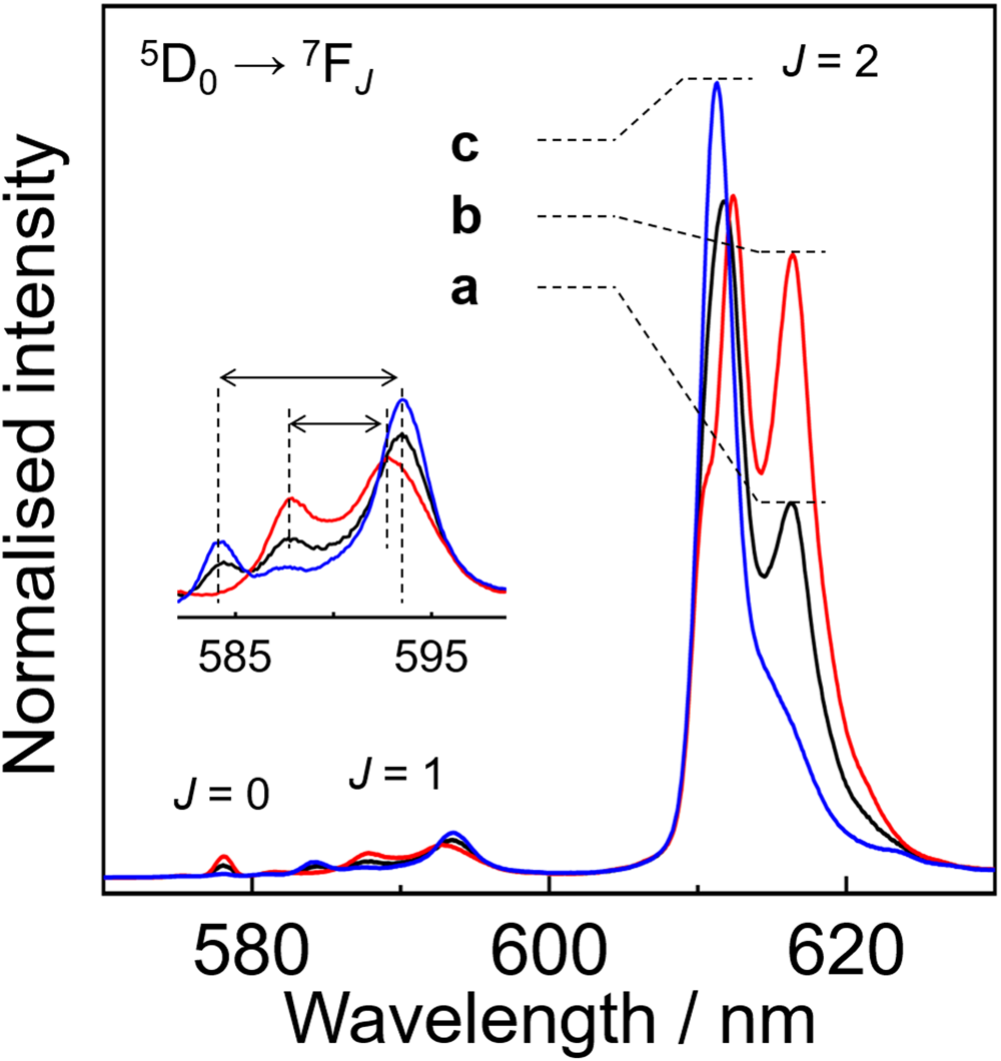
Photoluminescence spectra of (**a**) **Eu**(+)-Ex0 (1 × 10^−3^ M, black), (**b**) **Eu**(+)-Ex498 (1 × 10^−3^ M, red), and (**c**) **Eu**(+)-Ex0 (1 × 10^−5^ M, blue) excited at 350 nm in acetone.

Photophysical properties of Eu(III) complexes are affected by the coordination geometry^38^. The emission spectra of **Eu**(+)-Ex0 and -Ex498 in acetone (1 × 10^−3^ M) are shown in Fig. 3 (black; **a**, and red; **b**). The Eu(III) complexes show sharp emission peaks in the region of 570–630 nm, which are attributed to the ^5^D_0_ → ^7^F_*J*_ (*J* = 0, 1, and 2) transitions of Eu(III) ions. The spectra were normalised with respect to the integrated intensities of the magnetic dipole transition (^5^D_0_ → ^7^F_1_). Their spectral shapes in liquid media were different from that of [Eu(+tfc)_3_(tppo)_2_] in the solid state (Supplementary Fig. S1). The emission spectra for the ^5^D_0_ → ^7^F_1_ and ^5^D_0_ → ^7^F_2_ transitions were also changed in response to the concentration of the tppo molecules in solution. In particular, the ^5^D_0_ → ^7^F_1_ transition band is composed of three Stark sublevels under the electric field (crystal field). **Eu**(+)-Ex0 showed three peaks at 584.5, 588, and 593.5 nm in the ^5^D_0_ → ^7^F_1_ transition (Fig. 3 inset, black; **a**), whereas **Eu**(+)-Ex498 showed two peaks at 588 and 593 nm (Fig. 3 inset, red; **b**). The ^5^D_0_ → ^7^F_1_ transition of **Eu**(+)-Ex0 at a lower concentration (1 × 10^−5^ M, Fig. 3, blue; **c**) also showed three peaks at 584, 587.5, and 593.5 nm, which are similar to that of **Eu**(+)-Ex0 at a higher concentration. The small peak at 587.5 nm can be attributed to the ^5^D_1_ → ^7^F_3_ transition, which is sometimes observed in the same energy region as the ^5^D_0_ → ^7^F_1_ transition^38^. The emission bands at around 612 nm are attributed to hypersensitive electric dipole transitions (^5^D_0_ → ^7^F_2_), which are strongly dependent on the local symmetry of the Eu(III) ion. The change of spectral shape is influenced by the rearrangement of coordination geometries of the Eu(III) complex depending on additional tppo molecules. In case of the non-coordinating toluene solution, the emission spectra of **Eu**(+)-Ex0 and -Ex48 were similar in shape to that of **Eu**(+)-Ex498 in acetone, irrespective of the amount of additional tppo molecules (Supplementary Fig. S2). We propose that the inner coordination structure of **Eu**(+)-Ex498 in acetone is composed of one Eu(III) ion, three +tfc ligands, and two tppo ligands.

**Figure 4:**
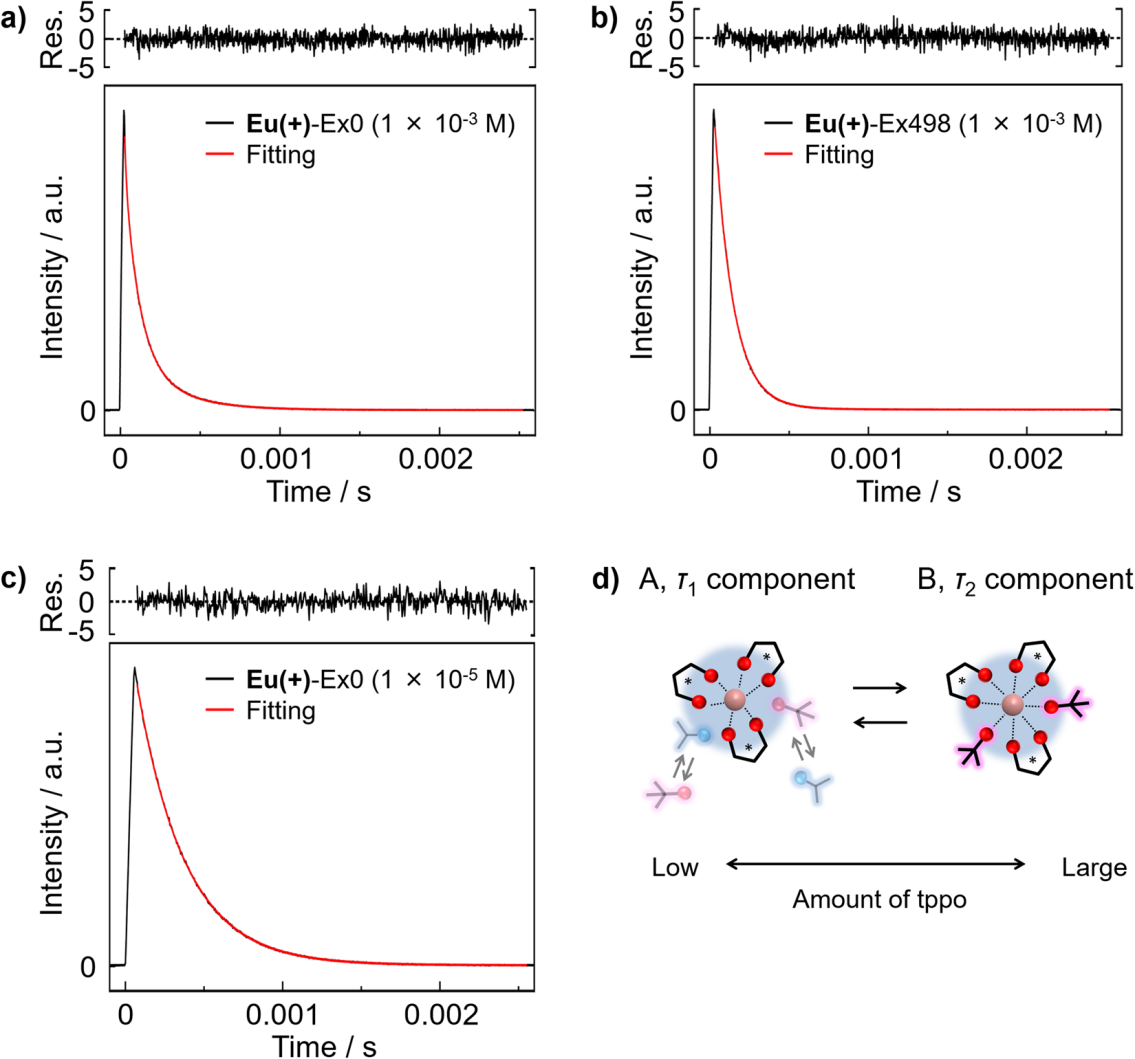
Emission decay profiles, fittings, and residuals (Res.) of (**a**) **Eu**(+)-Ex0 (1 × 10^−3^ M), (**b**) **Eu**(+)-Ex498 (1 × 10^−3^ M), and (**c**) **Eu**(+)-Ex0 (1 × 10^−5^ M) at 612 nm excited at 356 nm in acetone. (**d**) The coordination image of the *τ*_1_ and *τ*_2_ components of the Eu(III) complex in acetone.

The time-resolved emission profiles of **Eu**(+)-Ex0 and -Ex498 in acetone (1 × 10^−3^ M) were measured to clarify their coordination structures. The emission lifetimes were estimated using triple (for **Eu**(+)-Ex0) or double (for **Eu**(+)-Ex498) exponential functions to analyse several conformations in solution (Fig. 4a,b). The estimated emission lifetimes are summarised in Table 1. For **Eu**(+)-Ex0, the emission lifetimes (*τ*_1_, *τ*_2_, and *τ*_3_) and their ratios were calculated to be 0.27 ms (50%), 0.09 ms (49%), and 0.01 ms (1%), respectively. The longer component *τ*_1_ in higher concentration (1 × 10^−3^ M, 0.27 ms) was similar to the single component *τ*_1_ in lower concentration (1 × 10^−5^ M, 0.31 ms, Fig. 4c). We consider that the *τ*_1_ component is the Eu(III) complex with coordinating acetone molecules (Fig. 4a,d). The *τ*_1_ and *τ*_2_ component ratios decreased and increased, respectively, with increasing amount of tppo molecules. The main *τ*_2_ value of **Eu**(+)-Ex498 was found to be 0.12 ms (97%). The lifetime *τ*_2_ in acetone was similar to the single-lifetime component in toluene (Supplementary Table S2), indicating that the Eu(III) ion is attached with three +tfc and two tppo ligands (Fig. 4b,d). We revealed that the two types of steric structures with *τ*_1_ and *τ*_2_ components were reorganized in response to the external tppo and acetone molecules.

**Table 1:** Luminescence properties of **Eu**(+)-Ex*n* excited at 356 nm in acetone^a^.

	Concentration [M]	*τ*_1_ [ms]	*τ*_2_ [ms]	*τ*_3_ [ms]	*g*_CPL_
**Eu**(+)-Ex0	1 × 10^−3^	0.27 (50%)	0.09 (49%)	0.01 (1%)	−0.44
**Eu**(+)-Ex498	1 × 10^−3^	0.37 (3%)	0.12 (97%)	—	+0.013
**Eu**(+)-Ex0	1 × 10^−5^	0.31 (100%)	—	—	−1.0

^a^Emission decay curves were analysed by multi-exponential curve fittings $[I(t)={\sum }^{}{A}_{i}\exp (\,-\,t/{\tau }_{i})]$. The ratio of each component denotes $100\times {A}_{i}{\tau }_{i}/{\sum }^{}{A}_{i}{\tau }_{i}$.

### Chiroptical properties

**Figure 5:**
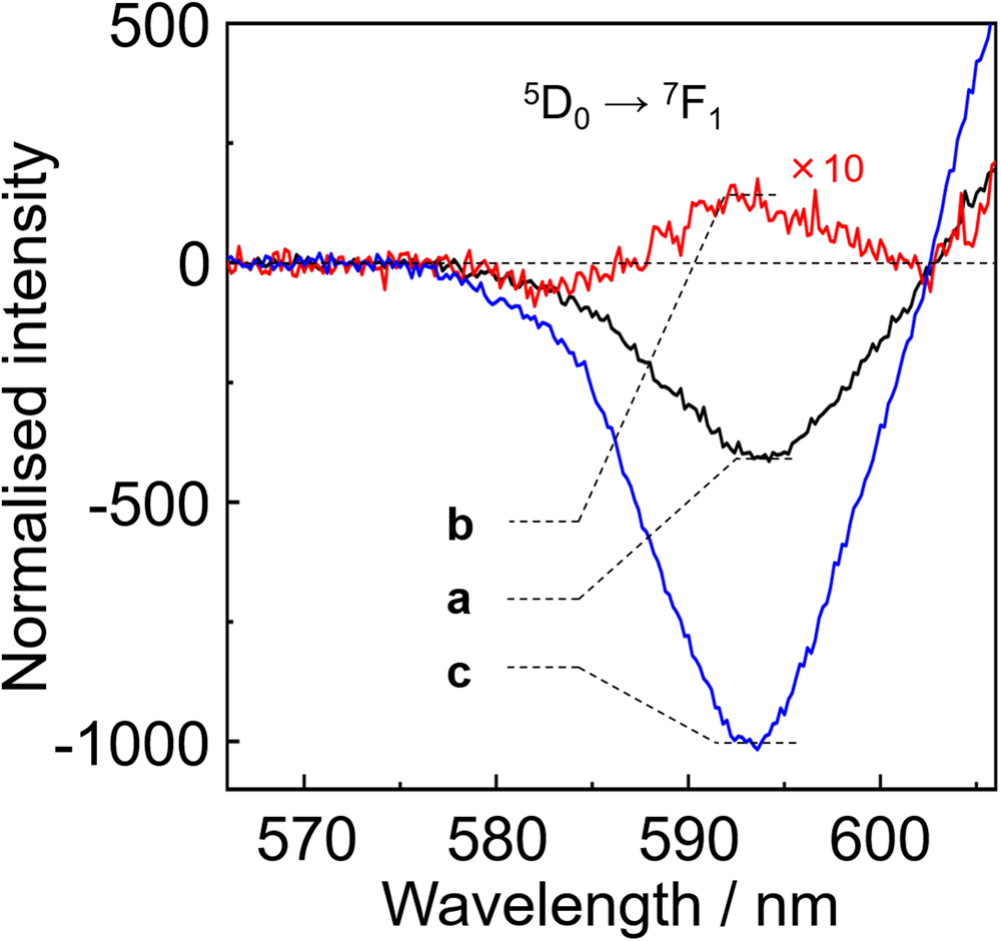
CPL spectra of (**a**) **Eu**(+)-Ex0 (1 × 10^−3^ M, black), (**b**) **Eu**(+)-Ex498 (1 × 10^−3^ M, red), and (**c**) **Eu**(+)-Ex0 (1 × 10^−5^ M, blue) excited at 350 nm in acetone.

The CPL spectra and dissymmetry factors of **Eu**(+)-Ex0 and -Ex498 are shown in Fig. 5 and Table 1, respectively. The CPL signals for the ^5^D_0_ → ^7^F_1_ transition were composed of two peaks at 583 and 594 nm. The CPL signals at 594 nm were inverted by the addition of tppo molecules. The CPL spectrum of **Eu**(+)-Ex0 (1 × 10^−3^ M) shows a large negative peak at 594 nm, the *g*_CPL_ value of which (−0.44) is similar to a previously reported *g*_CPL_ value (*g*_CPL_ = −0.47)^35^ in acetone-d_6_ (1 × 10^−3^ M). The CPL spectrum of **Eu**(+)-Ex498 (1 × 10^−3^ M, excess amount of tppo) exhibits a small positive peak (*g*_CPL_ = +0.013). The enantiomer [Eu(-tfc)_3_(tppo)_2_] also exhibits CPL sign inversion at the transition (Supplementary Fig. S3). The CPL intensity of **Eu**(+)-Ex0 in lower concentration (1 × 10^−5^ M, *g*_CPL_ = −1.0) is much larger than that in higher concentration (1 × 10^−3^ M, *g*_CPL_ = −0.44). In contrast, the CPL signals of **Eu**(+)-Ex0 and -Ex498 at around 583 nm in the ^5^D_0_ → ^7^F_1_ transition exhibit negative CPL signals in these conditions. The CPL sign inversion behaviours depending on the external environments are summarised in Supplementary Fig. S4. Law and Dai reported similar CPL sign inversion phenomena of chiral Eu(III) complexes in the ^5^D_0_ → ^7^F_1_ transition depending on solvents (Supplementary Fig. S4b)^39^.

Considering the presence of *τ*_1_ and *τ*_2_ components in the emission lifetime measurements, the observed CPL spectra of **Eu**(+)-Ex0 and -Ex498 in acetone (1 × 10^−3^ M) were attributed to several equilibrium states of the Eu(III) complex in acetone. The large negative *g*_CPL_ of **Eu**(+)-Ex0 is dominated by the *τ*_1_ component related to coordinating acetone molecules (Supplementary Figs S5–S7). The small positive *g*_CPL_ of **Eu**(+)-Ex498 is related to the *τ*_2_ component of the eight-coordinated Eu(III) complex with two inner tppo ligands; this was supported by the similar positive *g*_CPL_ (+0.036) in toluene (Supplementary Fig. S8).

## Discussion

**Figure 6:**
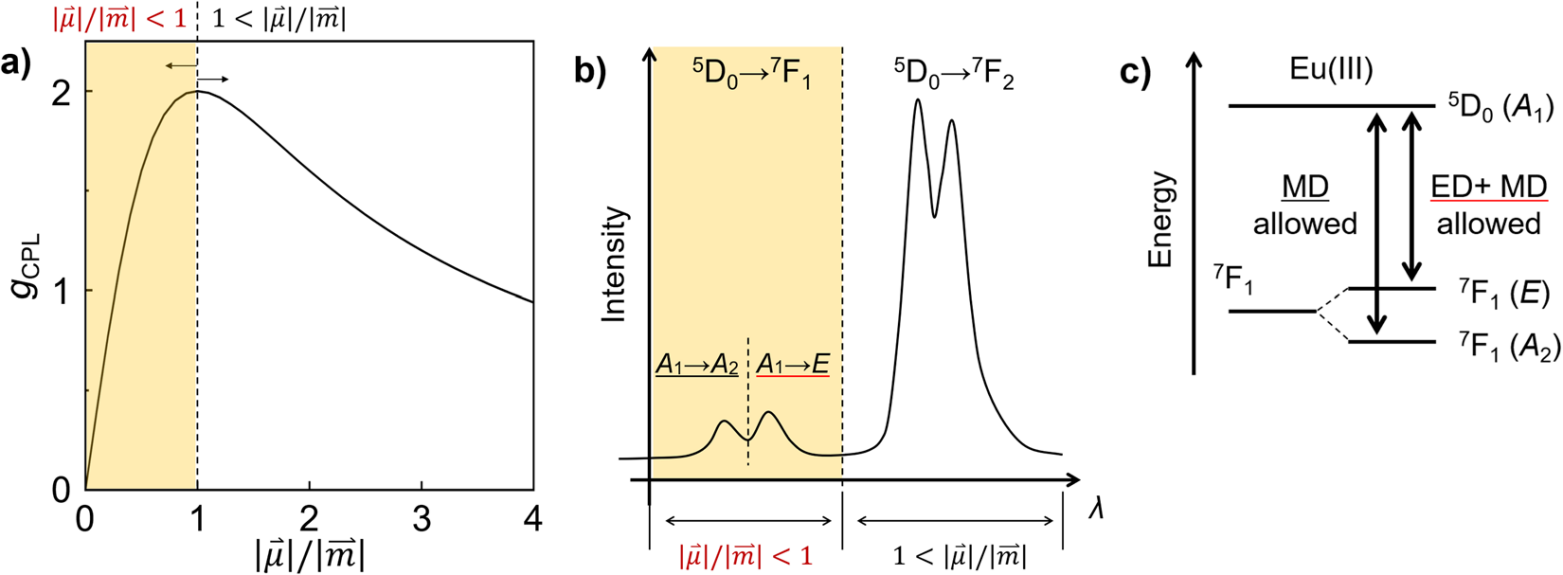
(**a**) A simulated *g*_CPL_ curve against $|\overrightarrow{\mu }|/|\overrightarrow{m}|$ ratio in equation (1) with *θ* = 0^°^. (**b**) An attribution of photoluminescence spectrum and (**c**) an energy diagram in the ^5^D_0_ → ^7^F_1_ transition of Eu(III) complex (C_4v_ or D_2d_) based on the electronic structure and group theory.

The dissymmetry factor *g*_CPL_ is expressed in terms of the transition electric dipole moment $\overrightarrow{\mu }$ and transition magnetic dipole moment $\overrightarrow{m}$ as follows^28^,

1 $${g}_{{\rm{CPL}}}=4\cdot \frac{|\overrightarrow{\mu }||\overrightarrow{m}|\cos \,\theta }{{|\overrightarrow{\mu }|}^{2}+{|\overrightarrow{m}|}^{2}}=4\cdot \frac{(\frac{|\overrightarrow{\mu }|}{|\overrightarrow{m}|})\cos \,\theta }{{(\frac{|\overrightarrow{\mu }|}{|\overrightarrow{m}|})}^{2}+1},$$

where *θ* is the angle between $\overrightarrow{\mu }$ and $\overrightarrow{m}$. When $\overrightarrow{\mu }=\overrightarrow{m}\,(\theta ={0}^{^\circ })$, equation (1) provides the largest *g*_CPL_ value (=2) mathematically (Fig. 6a). In the region $|\overrightarrow{\mu }|/|\overrightarrow{m}| < 1$ (Fig. 6a,b, orange regions), the Eu(III) complex with a large $|\overrightarrow{\mu }|$ provides a large *g*_CPL_ value. In general, the $|\overrightarrow{m}|$ value in the ^5^D_0_ → ^7^F_1_ transition is larger than the $|\overrightarrow{\mu }|$ value ($|\overrightarrow{\mu }|/|\overrightarrow{m}| < 1$)^40^. The intensity of $|\overrightarrow{\mu }|$ in the ^5^D_0_ → ^7^F_1_ transition depends on the crystal field around the Eu(III) ion^41,42^. The ^7^F_1_ energy level of the Eu(III) ion in a typical eight-coordinate structure (C_4v_ or D_2d_) splits into two Stark sublevels (Fig. 6b)^38^. The two bands at 583 and 594 nm in the CPL spectra are assigned to the *A*_1_ → *A*_2_ and *A*_1_ → *E* transitions^42^, respectively, in Fig. 6b,c. The observed CPL signal in the *A*_1_ → *E* transition was inverted from minus to plus, while that in the *A*_1_ → *A*_2_ transition retained the minus sign. In C_4v_ or D_2d_ symmetry, the direct product *A*_2_ (=*A*_1_ × *A*_2_) is expressed in terms of the electric dipole (ED) forbidden and magnetic dipole (MD) allowed transitions (*R*_*z*_) on the character table in group theory (Fig. 6c, Supplementary Tables S3 and S4). On the other hand, the direct product *E* (=*A*_1_ × *E*) produces ED and MD allowed transitions ((*x*, *y*); (*R*_x_, *R*_y_), Fig. 6c, Supplementary Tables S3 and S4). The CPL sign at 583 nm (ED forbidden *A*_1_ → *A*_2_ transition) reflects the intrinsic Λ- or Δ-type structure, because of insensitive electronic state mixing. Considering the same CPL sign at 583 nm of the *τ*_1_ and *τ*_2_ components, the structure-type (Λ or Δ) with the *τ*_1_ component is the same as that with the *τ*_2_ component in our experiments. The coordination symmetry of *τ*_1_ component was much similar to that of *τ*_2_ component, which was supported by magnetic circular dichroism (MCD) measurements and DFT calculation (Supplementary Figs S9–S11). In contrast, the CPL sign in the ED and MD allowed *A*_1_ → *E* transition is sensitive to electronic state mixing even for the same chiral structure-type (Λ or Δ). The effective sign inversion and drastic intensity change of the CPL signal in the *A*_1_ → *E* transition should be caused by the change of $\overrightarrow{\mu }$ based on electronic state mixing.

In the ^5^D_0_ → ^7^F_1_ transition, $\overrightarrow{\mu }$ is mainly altered by the *J*-mixing of ^7^F_2_ or ^7^F_3_ sublevels into ^7^F_1_^38,43^. In the photoluminescence spectra, the Stark splitting energy of the *τ*_1_ component (270 cm^−1^, Fig. 3 inset, blue; **c**) was larger than that of the *τ*_2_ component (160 cm^−1^, Fig. 3 inset, red; **b**). The large Stark splitting energy suggests the large *J*-mixing in the *A*_1_ → *E* transition of the *τ*_1_ component^43^. The *J*-mixing increases the $|\overrightarrow{\mu }|$ value at the ED allowed *A*_1_ → *E* transition relative to that at the ED forbidden *A*_1_ → *A*_2_ transition, which is consistent with relatively large emission intensity at the *A*_1_ → *E* transition (593.5 nm) of the *τ*_1_ component (Fig. 3 inset, blue; **c**). The $|\overrightarrow{\mu }|$ increase leads to the large *g*_CPL_ value in equation (1).

The angle *θ* between $\overrightarrow{\mu }$ and $\overrightarrow{m}$ of the *τ*_1_ component is larger than 90°, whereas that of the *τ*_2_ component is smaller than 90°, suggesting that the angle is established by $\overrightarrow{\mu }$ vector change due to *J*-mixing. The extra-large enhancement of *g*_CPL_ from +0.013 to −1.0 also indicates that *J*-mixing promotes the direction of $\overrightarrow{\mu }$ and $\overrightarrow{m}$ to antiparallel, leading to the large *g*_CPL_. We demonstrated that the CPL sign and intensity are strongly influenced by the chiral electronic structure depending on the $\overrightarrow{\mu }$ under *J*-mixing in the same chiral structure-type.

## Conclusion

We successfully observed a CPL sign inversion with a drastic *g*_CPL_ change from +0.013 to −1.0 for the Eu(III) complex with the same chiral coordination structure-type. The CPL phenomena were attributed to the chiral electronic structure depending on the $\overrightarrow{\mu }$ under *J*-mixing. We also achieved an extra-large *g*_CPL_ (−1.3) of the Eu(III) complex with chiral +tfc ligands in DMSO, suggesting that the *g*_CPL_ of the Eu(III) complex could be enhanced by *J*-mixing with a small 4f-5d mixing character (Supplementary Figs S12 and S13, Table S5). The results provide a novel aspect for the optical activity of metal complexes and molecular design of chiral lanthanide complex by maximising the *g*_CPL_ value.

## Methods

### Synthesis of Tris(3-trifluoroacetyl-(+)-camphorato)europium(III) bis(triphenylphosphine oxide) ([Eu(+tfc)_3_(tppo)_2_])

Europium(III) acetate *n*-hydrate (0.36 g) was dissolved in distilled water (150 mL), and a few drops of 28% ammonia solution were added. (+)-3-triflouroacetyl camphor (+tfc, 0.50 g, 2.0 mmol) in methanol (20 mL) was added to the solution, and the mixture was stirred for 3 h at room temperature. The obtained powder was washed with distilled water, and the powder was dried in vacuo (0.42 g). The powder (0.19 g) and triphenylphosphine oxide (tppo, 0.11 g, 0.40 mmol) were dissolved in methanol (20 mL) and refluxed for 3 h. The reaction solution was evaporated using a rotary evaporator. The obtained powder was recrystallised from a hot acetonitrile solution and gave yellow crystals. Yield: 47%. Elemental analysis: Calculated for C_72_H_72_EuF_9_O_8_P_2_: C, 59.63%, H, 5.00%. Found: C, 59.54%, H, 4.92%^35^.

### Synthesis of Tris(3-trifluoroacetyl-(−)-camphorato)europium(III) bis(triphenylphosphine oxide) ([Eu(-tfc)_3_(tppo)_2_])

[Eu(-tfc)_3_(tppo)_2_] was prepared using the same method for [Eu(+tfc)_3_(tppo)_2_], starting from (−)-3-triflouroacetyl camphor, yielding yellow powder. Yield: 64%. Elemental analysis: Calculated for C_72_H_72_EuF_9_O_8_P_2_: C, 59.63%, H, 5.00%. Found: C, 59.49%, H, 4.94%.

## Materials

Europium(III) acetate *n*-hydrate, acetone-d_6_ (99.9%), acetone (spectroscopic grade), toluene (spectroscopic grade), dimethyl sulfoxide (spectroscopic grade), and 28% ammonia solution were purchased from Wako Pure Chemical Industries Ltd. (+)-3-(trifluoroacetyl)camphor and (−)-3-(trifluoroacetyl)camphor were purchased from Sigma-Aldrich Co. Triphenylphosphine oxide was purchased from Tokyo Chemical Industry Co., Ltd. All other chemicals and solvents were of reagent grade and were used without further purification.

### Apparatus

Elemental analyses were performed on an Exeter Analytical CE440. Proton nuclear magnetic resonance (^1^H NMR) spectra were recorded in acetone-d_6_ on an auto-NMR JEOL ECS 400 MHz; Acetone (δ_H_ = 2.05 ppm) was used as an internal reference. Emission spectra and emission lifetimes were measured using a Horiba/Jobin-Yvon FluoroLog-3 spectrofluorometer. CPL spectra were measured using a JASCO CPL-200 spectrofluoropolarimeter.

## Electronic supplementary material

Supplementary Information
