# Supplementary material for: Electronic chirality inversion of lanthanide complex induced by achiral molecules
Source: Sci Rep. 2018 Nov 6;8:16395. doi: 10.1038/s41598-018-34790-0 (PMC6219555; doi:10.1038/s41598-018-34790-0)
Supplement: Supplementary file 1 — Supplementary Information [file 41598_2018_34790_MOESM1_ESM.docx]

Supplementary Information

Electronic chirality inversion of lanthanide complex induced by achiral molecules

Satoshi Wada^1^, Yuichi Kitagawa^2^, Takayuki Nakanishi^3^, Masayuki Gon^4^, Kazuo Tanaka^4^, Koji Fushimi^2^, Yoshiki Chujo^4^ & Yasuchika Hasegawa^2^

^1^Graduate School of Chemical Sciences and Engineering, Hokkaido University, N13 W8, Kita-ku, Sapporo, Hokkaido 060–8628, Japan

^2^Faculty of Engineering, Hokkaido University, N13 W8, Kita-ku, Sapporo, Hokkaido 060–8628, Japan

^3^Faculty of Industrial Science and Technology, Tokyo University of Science, 6-3-1 Niijuku, Katsushika-ku, Tokyo 125-8585, Japan

^4^Graduate School of Engineering, Kyoto University, Katsura, Nishikyo-ku, Kyoto 615-8510, Japan

Correspondence and requests for materials should be addressed to Y.K. (email: y-kitagawa@eng.hokudai.ac.jp) or Y.H. (email: hasegaway@eng.hokudai.ac.jp)

S1 Photoluminescence spectrum


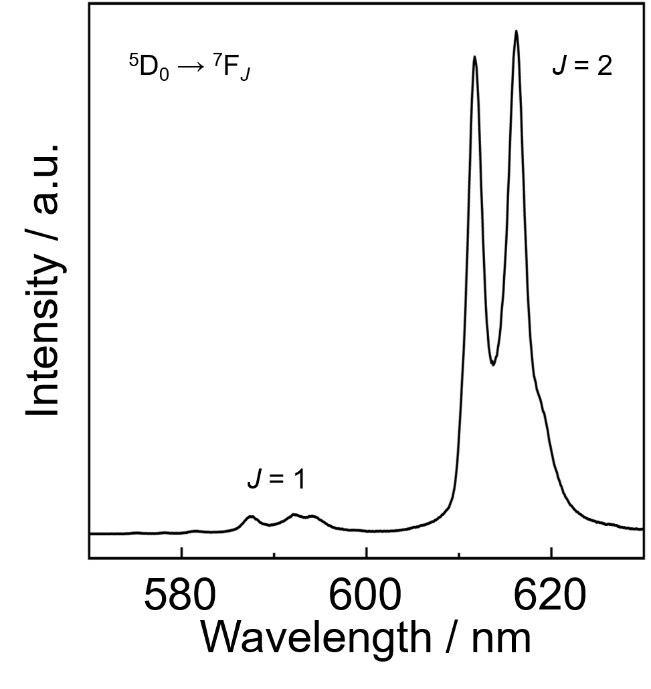


**Figure S1.** Photoluminescence spectrum of **Eu(+)** excited at 350 nm in powder.

S2 Photoluminescence spectra


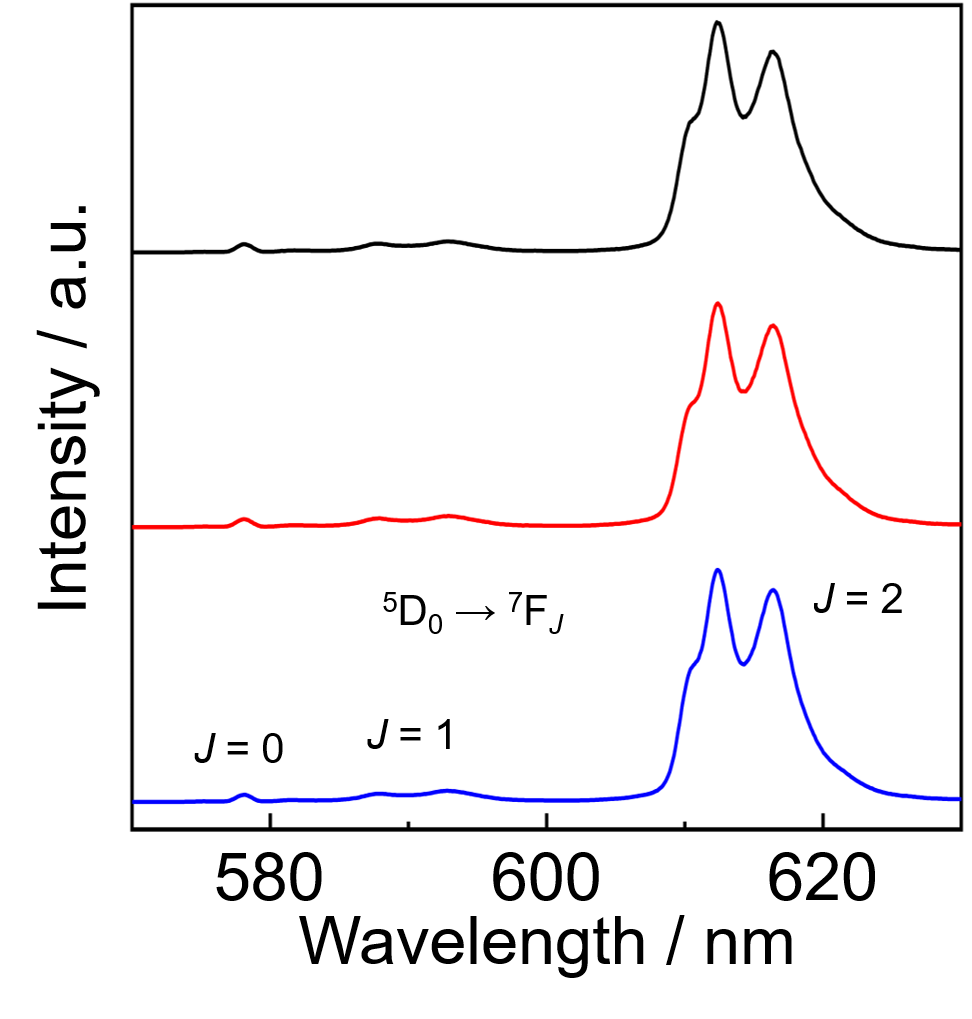


**Figure S2.** Photoluminescence spectra of **Eu(+)**-Ex0 (1 × 10^-3^ M, black) in toluene, **Eu(+)**-Ex48 (1 × 10^-3^ M, red) in toluene, and **Eu(+)**-Ex498 (1 × 10^-3^ M, blue) in acetone, excited at 350 nm.

S3 CPL spectra


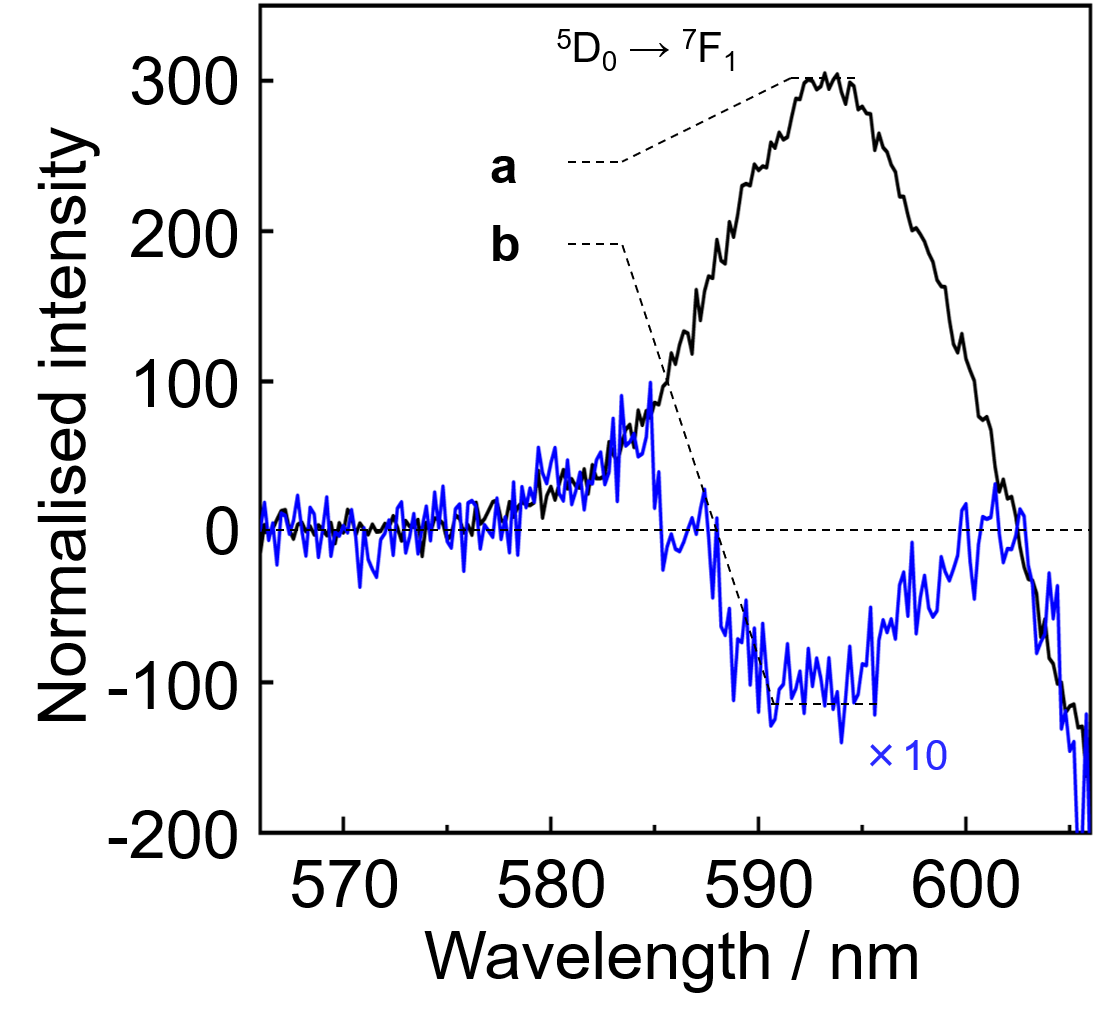


**Figure S3.** CPL spectra of [Eu(-tfc)_3_(tppo)_2_] (**a**) without tppo addition (1 × 10^-3^ M, black) and (**b**) with additional 498 equivalents of tppo (1 × 10^-3^ M, blue) in acetone, excited at 350 nm.

S4 CPL images


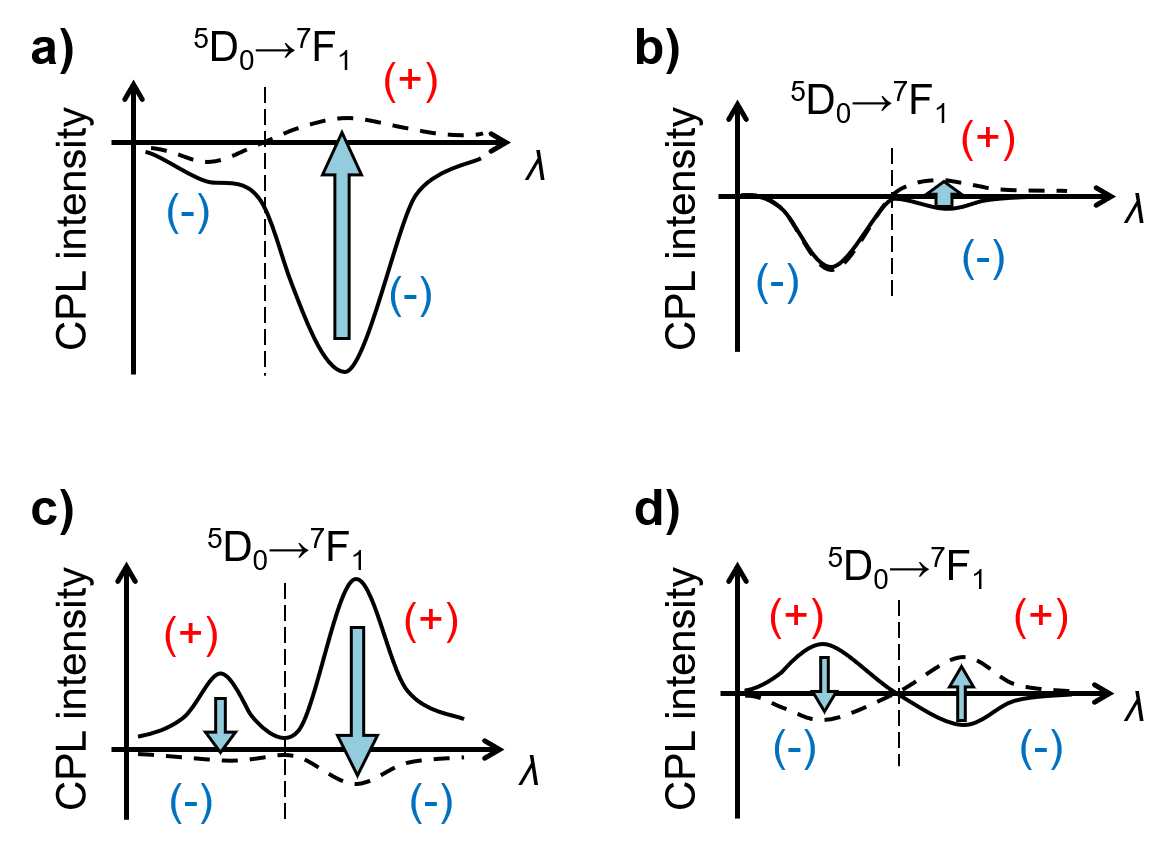


**Figure S4.** Graphical images of CPL sign inversion in the ^5^D_0_ → ^7^F_1_ transition reported by (**a**) our experiment, (**b**) Law^1^, (**c**) Yuasa^2^, and (**d**) Parker^3^.

S5 Emission and CPL spectra

As a Eu(III) complex without tppo molecules, Eu(+tfc)_3_(H_2_O)_2_ in acetone (1 × 10^-3^ M) were prepared. The emission and CPL spectra of Eu(+tfc)_3_(H_2_O)_2_ in acetone (1 × 10^-3^ M) are shown in Figs. S5 and S6. The emission spectral shape agreed with that of **Eu(+)**-Ex0 in lower concentration (1 × 10^-5^ M, Fig. 3, blue; **c**), indicating their coordination structures are similar to the *τ*_1_ component. The time-resolved emission profile exhibited single exponential decay for the Eu(+tfc)_3_(H_2_O)_2_ in acetone (1 × 10^-3^ M). The Eu(+tfc)_3_(H_2_O)_2_ exhibited negatively large CPL signal (*g*_CPL_ = -0.97) in the ^5^D_0_ → ^7^F_1_ transition as well as that of **Eu(+)**-Ex0 in lower concentration (1 × 10^-5^ M, *g*_CPL_ = -1.0, Fig. 5, blue; **c**). These results support that the large *g*_CPL_ value of *τ*_1_ component is related to acetone molecules around the Eu(III) ion.

| 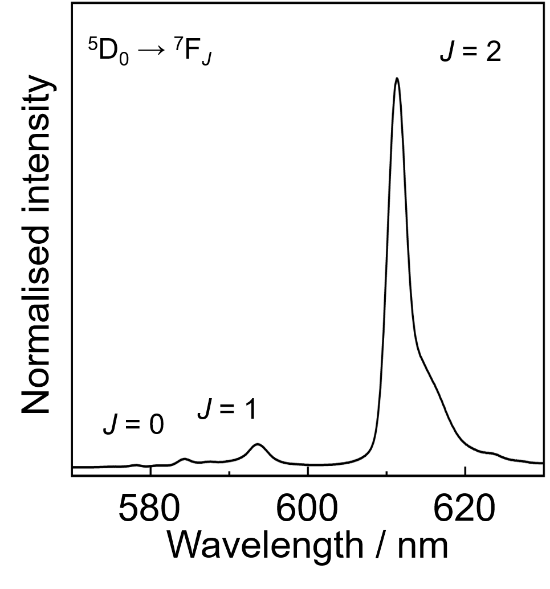 | 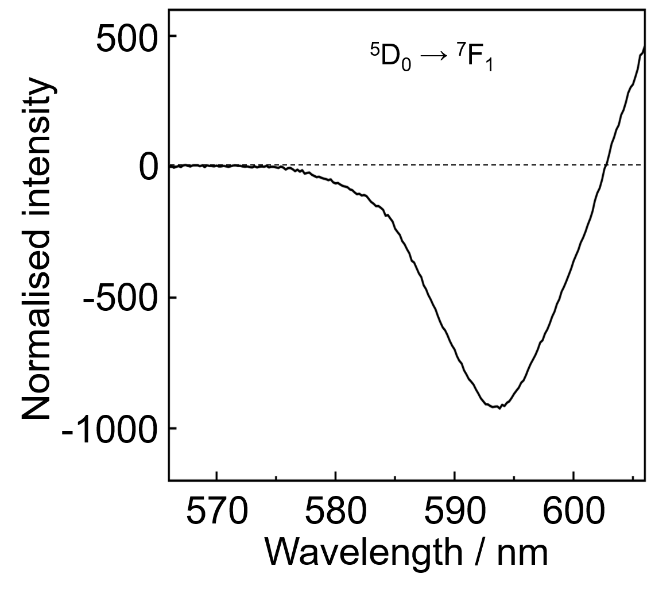 |
| --- | --- |
| **Figure S5.** Emission spectrum of Eu(+tfc)_3_(H_2_O)_2_ in acetone (1 × 10^-3^ M), excited at 350 nm. | **Figure S6.** CPL spectrum of Eu(+tfc)_3_(H_2_O)_2_ in acetone (1 × 10^-3^ M), excited at 350 nm. |

S6 Photoluminescence spectra

The emission spectrum of Eu(+tfc)_3_(H_2_O)_2_ in CHCl_3_ (1 × 10^-3^ M), Eu(+tfc)_3_(H_2_O)_2_ in CHCl_3_ (1 × 10^-3^ M) with 100 equivalents of acetone molecules, and Eu(+tfc)_3_(H_2_O)_2_ in acetone (1 × 10^-3^ M) are shown in Fig. S7 to clarify the coordination geometry around Eu(III) ion in *τ*_1_ component. The emission spectrum of Eu(+tfc)_3_(H_2_O)_2_ in CHCl_3_ (1 × 10^-3^ M, Fig. S7, black; **a**) was similar to that of Eu(+tfc)_3_(H_2_O)_2_ in CHCl_3_ (1 × 10^-3^ M) with 100 equivalents of acetone molecules (Fig. S7, red; **b**). In contrast, these spectra of Eu(+tfc)_3_(H_2_O)_2_ in CHCl_3_ were different from that of Eu(+tfc)_3_(H_2_O)_2_ in acetone (1 × 10^-3^ M, Fig. S7, blue; **c**). The results support that the coordination geometry around Eu(III) ion of Eu(+tfc)_3_(H_2_O)_2_ in CHCl_3_ is different from that in acetone.


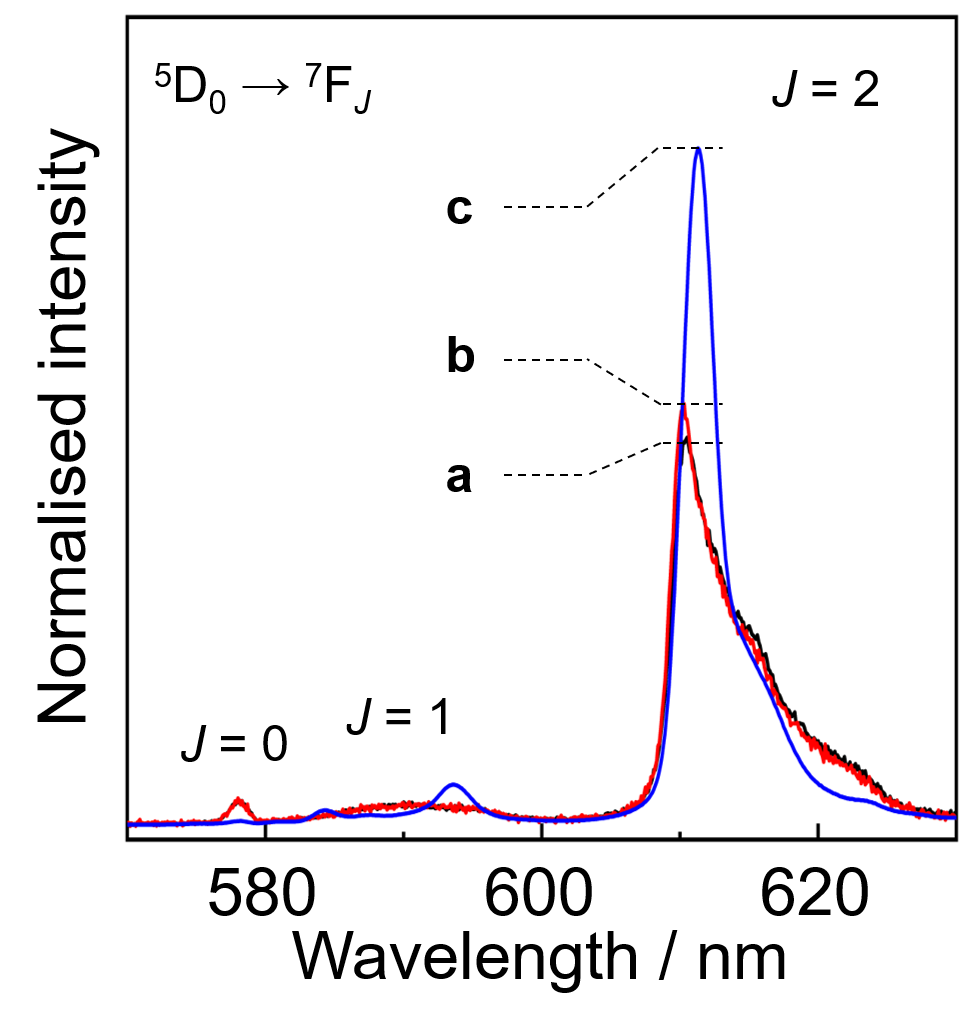


**Figure S7.** Emission spectra of (**a**) Eu(+tfc)_3_(H_2_O)_2_ in CHCl_3_ (1 × 10^-3^ M, black), (**b**) Eu(+tfc)_3_(H_2_O)_2_ in CHCl_3_ (1 × 10^-3^ M) with 100 equivalents of acetone molecules (red), and (**c**) Eu(+tfc)_3_(H_2_O)_2_ in acetone (1 × 10^-3^ M, blue), excited at 350 nm.

S7 CPL spectrum


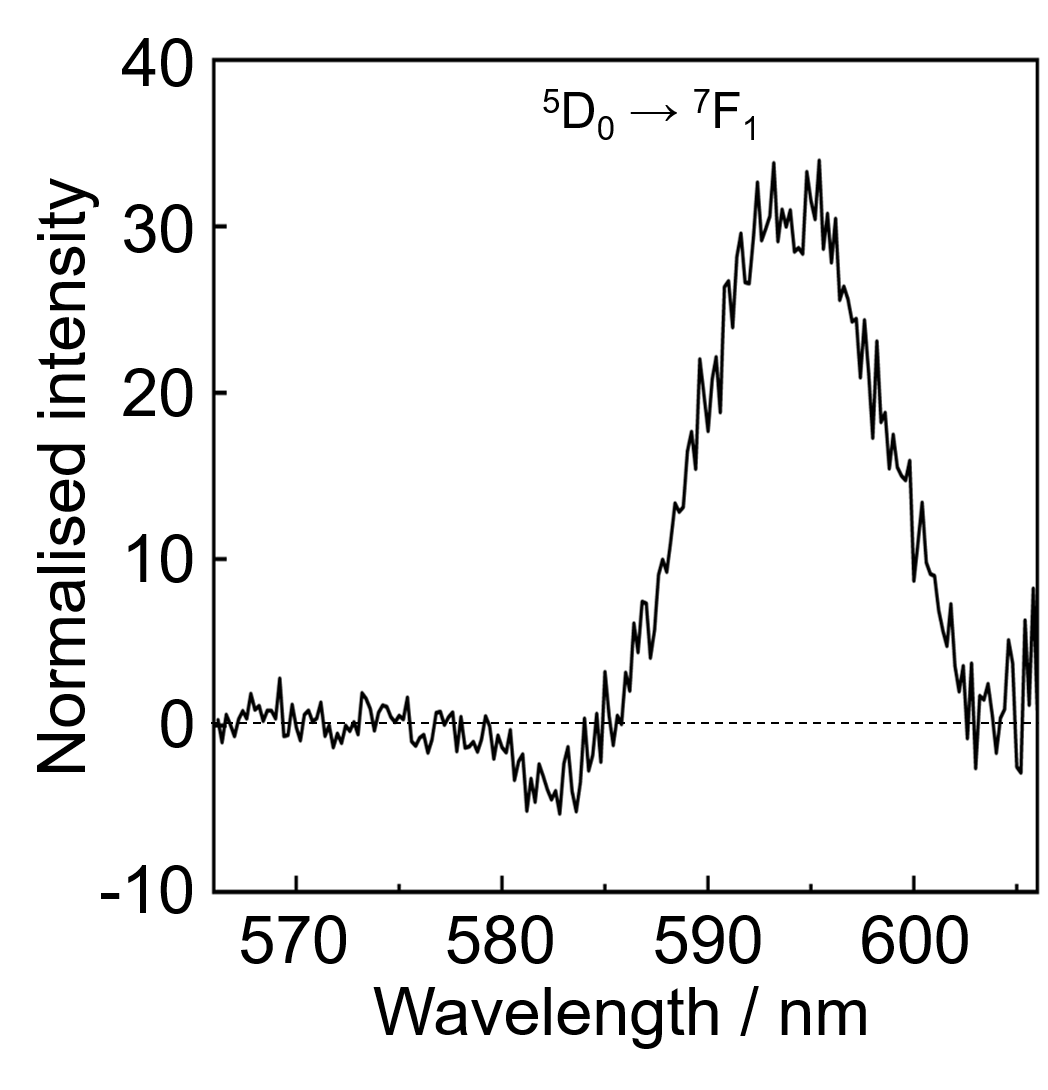


**Figure S8.** CPL spectrum of **Eu(+)**-Ex48 (1 × 10^-3^ M) in toluene, excited at 350 nm.

S8 Emission and CPL spectra

MCD spectra of Eu(III) complexes were measured for more information about the coordination structure. Eu(+tfc)_3_(tppo)_2_ with 28 equivalents of tppo (**Eu(+)**-Ex28) and Eu(+tfc)_3_(H_2_O)_2_ in acetone with high concentration (1 × 10^-2^ M) were prepared. In high concentration (1 × 10^-2^ M), the 28 equivalents of tppo molecules keep the coordination structure of Eu(+tfc)_3_(tppo)_2_ in acetone. The time-resolved emission profiles exhibited single exponential decay for these complexes in acetone. The emission, MCD, and absorption spectra of these complexes are shown in Figs. S9 and S10. The emission spectral shapes of **Eu(+)**-Ex28 and Eu(+tfc)_3_(H_2_O)_2_ in acetone agreed with those of **Eu(+)**-Ex498 in acetone (1 × 10^-3^ M, Fig. 3, red; **b**) with τ_2_ component and **Eu(+)**-Ex0 in acetone (1 × 10^-5^ M, Fig. 3, blue; **c**) with τ_1_ component, respectively. The MCD spectra of these Eu(III) complexes exhibited a negative *A* term in the electric dipole transition (^5^D_2_ ← ^7^F_0_). The sign of *A* term in the MCD spectrum depends on the symmetry of the first coordination sphere around the Eu(III) ion^4^. A typical eight-coordinated Eu(III) complex with distorted square antiprism (SAP, C_4v_) and dodecahedron (DH, D_2d_) exhibit a positive and negative *A* term in the ^5^D_2_ ← ^7^F_0_ transition, respectively^4,5^. The observed negative *A* terms indicate the coordination geometries of these complexes are related to DH-like structures. The MCD intensity of **Eu(+)**-Ex28 was also nearly equal to that of Eu(+tfc)_3_(H_2_O)_2_, indicating the similar coordination structure type for **Eu(+)**-Ex28 and Eu(+tfc)_3_(H_2_O)_2_ in acetone.

| 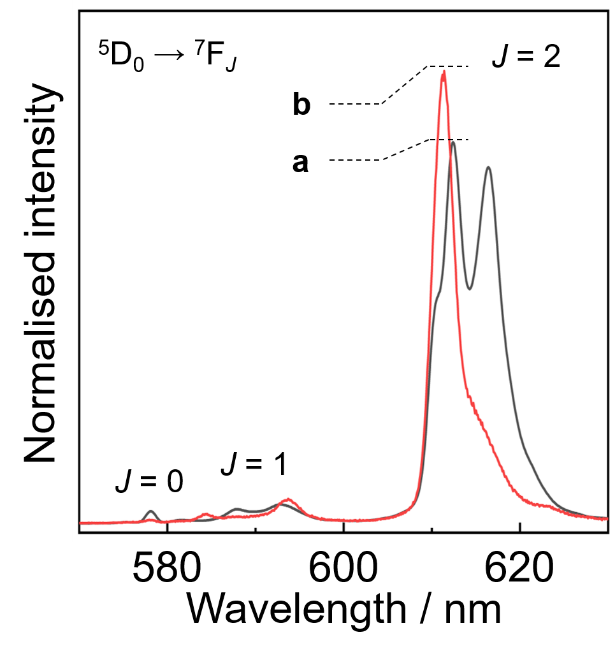 | 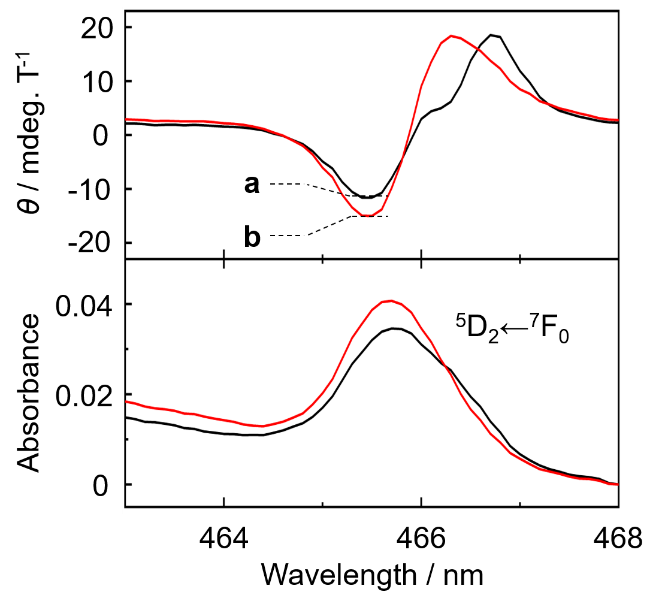 |
| --- | --- |
| **Figure S9.** Emission spectra of (**a**) **Eu(+)**-Ex28 (1 × 10^-2^ M, black) and (**b**) Eu(+tfc)_3_(H_2_O)_2_ (1 × 10^-2^ M, red) in acetone, excited at 400 nm. | **Figure S10.** (top) MCD and (bottom) absorption spectra of (**a**) **Eu(+)**-Ex28 (1 × 10^-2^ M, black) and (**b**) Eu(+tfc)_3_(H_2_O)_2_ (1 × 10^-2^ M, red) in acetone. |

S9 DFT calculation

DFT calculation of a simplified complex model, an yttrium(Y(III)) complex with camphor and acetone molecules, was performed. The X-ray crystal structure of Eu(+tfc)_3_(tppo)_2_ was chosen for the initial structure^6^, and tppo molecules were replaced by acetone molecules. The geometry optimisation was carried out using the DFT (B3LYP/LanL2DZ) method. The optimised geometry is shown in Fig. S11. The optimised coordination geometry of Y(III) complex with acetone molecules showed Δ-type structure, which is the same as the X-ray crystal structure of Eu(+tfc)_3_(tppo)_2_. The result supports the coordination structure type of Eu(III) complex with coordinating acetone molecules is similar to that of Eu(+tfc)_3_(tppo)_2_.


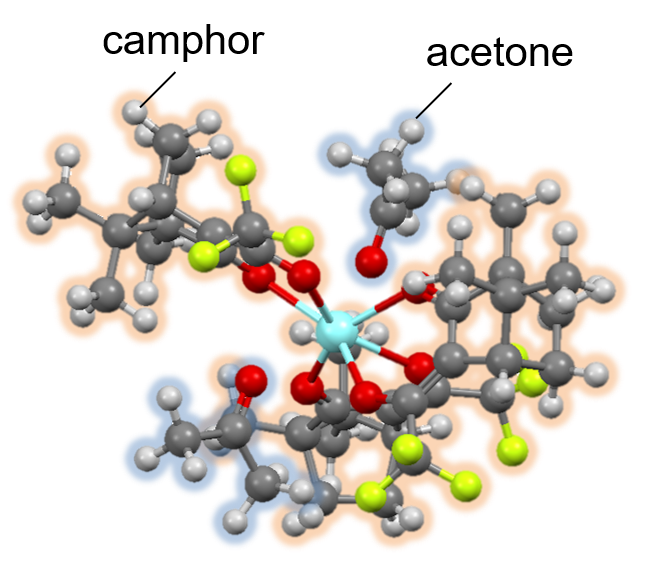


**Figure S11.** Optimised coordination structure of the Y(III) complex with three camphor ligands and two acetone molecules.

S10 Photoluminescence and CPL spectra

The emission spectrum of **Eu(+)**-Ex0 in DMSO (1 × 10^-3^ M, purple) provides the same splitting shape to that of **Eu(+)**-Ex0 in acetone (1 × 10^-5^ M, blue), as shown in Fig. S12. The result indicates that the coordination structure of **Eu(+)**-Ex0 in DMSO is similar to that of **Eu(+)**-Ex0 in acetone (1 × 10^-5^ M). The hypersensitive ^5^D_0_ → ^7^F_2_ transition of **Eu(+)**-Ex0 in DMSO is smaller than that of **Eu(+)**-Ex0 in acetone, indicating the small contribution of 4f-5d mixing in DMSO. The CPL of **Eu(+)**-Ex0 in DMSO (*g*_CPL_ = -1.3, Fig. S13, purple; **b**) is negatively larger in the ^5^D_0_ → ^7^F_1_ transition than that in acetone (1 × 10^-5^ M, Fig. S13, blue; **a**). These results imply that the extra-large *g*_CPL_ in DMSO may be attributed to the large *J*-mixing with small 4f-5d mixing character.

| 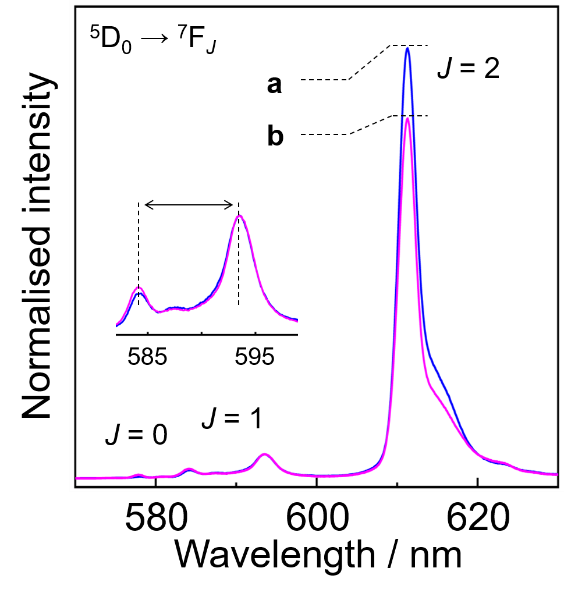 | 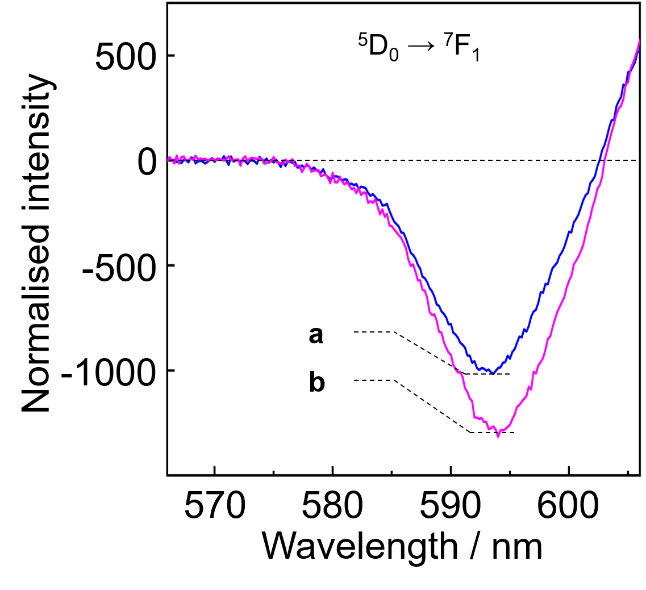 |
| --- | --- |
| **Figure S12.** Photoluminescence spectra of (**a**) **Eu(+)**-Ex0 in acetone (1 × 10^-5^ M, blue) and (**b**) in DMSO (1 × 10^-3^ M, purple), excited at 350 nm. | **Figure S13.** CPL spectra of **Eu(+)**-Ex0 (**a**) in acetone (1 × 10^-5^ M, blue) and (**b**) in DMSO (1 × 10^-3^ M, purple), excited at 350 nm. |

**Table S1.** ^1^H NMR peaks of **Eu(+)**-Ex*n* in acetone-d_6_ (**Eu(+)**; 1 × 10^-3^ M).

|  | A  [ppm] | B  [ppm] | C  [ppm] | D  [ppm] | E  [ppm] | F  [ppm] | G  [ppm] | H  [ppm] | I  [ppm] |
| --- | --- | --- | --- | --- | --- | --- | --- | --- | --- |
| **Eu(+)**-Ex0 | 7.87 | 1.35 | 0.68 | -0.07 | -0.21 | -0.39 | -1.19 | -1.40 | -2.09 |
| **Eu(+)**-Ex8 | 7.64 | 1.01 | 0.62 | -0.08 | 0.06 | -0.33 | -0.68 | -1.38 | -1.45 |
| **Eu(+)**-Ex28 | 7.63 | 0.92 | 0.60 | -0.07 | 0.14 | -0.31 | -0.54 | -1.37 | -1.30 |
| **Eu(+)**-Ex48 | 7.62 | 0.91 | 0.60 | -0.07 | 0.16 | -0.30 | -0.52 | -1.37 | -1.27 |
| **Eu(+)**-Ex98 | 7.62 | 0.90 | 0.60 | -0.06 | 0.17 | -0.30 | -0.49 | -1.37 | -1.24 |

**Table S2.** Luminescence properties of **Eu(+)**-Ex*n* excited at 356 nm in toluene.

|  | Concentration [M] | *τ* [ms] |
| --- | --- | --- |
| **Eu(+)**-Ex0 | 1 × 10^-3^ | 0.09  (100%) |
| **Eu(+)**-Ex48 | 1 × 10^-3^ | 0.13  (100%) |

**Table S3.** The character table for point group *C*_4_*_v_*.

| *C*_4_*_v_* | *E* | 2*C*_4_ | *C*_2_ | 2*σ_v_* | 2*σ_d_* |  | |
| --- | --- | --- | --- | --- | --- | --- | --- |
| *A*_1_ | 1 | 1 | 1 | 1 | 1 | *z* | *x*^2^ + *y*^2^, *z*^2^ |
| *A*_2_ | 1 | 1 | 1 | -1 | -1 | *R_z_* |  |
| *B*_1_ | 1 | -1 | 1 | 1 | -1 |  | *x*^2^ - *y*^2^ |
| *B*_2_ | 1 | -1 | 1 | -1 | 1 |  | *xy* |
| *E* | 2 | 0 | -2 | 0 | 0 | (*x*, *y*) ; (*R_x_*, *R_y_*) | (*xz*, *yz*) |

**Table S4.** The character table for point group *D*_2_*_d_*.

| *D*_2_*_d_* | *E* | 2*S*_4_ | *C*_2_ | 2*C*_2_^’^ | 2*σ_d_* |  | |
| --- | --- | --- | --- | --- | --- | --- | --- |
| *A*_1_ | 1 | 1 | 1 | 1 | 1 | *z* | *x*^2^ + *y*^2^, *z*^2^ |
| *A*_2_ | 1 | 1 | 1 | -1 | -1 | *R_z_* |  |
| *B*_1_ | 1 | -1 | 1 | 1 | -1 |  | *x*^2^ - *y*^2^ |
| *B*_2_ | 1 | -1 | 1 | -1 | 1 |  | *xy* |
| *E* | 2 | 0 | -2 | 0 | 0 | (*x*, *y*) ; (*R_x_*, *R_y_*) | (*xz*, *yz*) |

**Table S5.** Luminescence property of **Eu(+)**-Ex0 excited at 356 nm in DMSO^a^.

|  | Concentration [M] | *τ*_1_ [ms] | *τ*_2_ [ms] | *τ*_3_ [ms] | *g*_CPL_ |
| --- | --- | --- | --- | --- | --- |
| **Eu(+)**-Ex0 | 1 × 10^-3^ | 0.20  (91%) | 0.08  (3%) | 0.02  (6%) | -1.3 |

^a^ The emission decay curve was analysed by multi-exponential curve fitting [$I(t)=\sum A_{i}exp(-t/\tau_{i})$]. The ratio of each component denotes $100\times A_{i}\tau_{i}/\sum A_{i}\tau_{i}$.

**References**

1. Dai, L., Lo, W. S., Coates, I. D., Pal, R. & Law, G. L. New class of bright and highly stable chiral cyclen europium complexes for circularly polarized luminescence applications. *Inorg. Chem.* **55,** 9065–9070 (2016).

2. Yuasa, J., Ueno, H. & Kawai, T. Sign reversal of a large circularly polarized luminescence signal by the twisting motion of a bidentate ligand. *Chem. Eur. J.* **20,** 8621–8627 (2014).

3. Smith, D. G., Pal, R. & Parker, D. Measuring equilibrium bicarbonate concentrations directly in cellular mitochondria and in human serum using europium/terbium emission intensity ratios. *Chem. Eur. J.* **18,** 11604–11613 (2012).

4. Binnemans, K. Interpretation of europium(III) spectra. *Coord. Chem. Rev.* **295,** 1–45 (2015).

5. Görller-Walrand, C. Eu^3+^ as an MCD probe? *Chem. Phys. Lett.* **115,** 333–334 (1985).

6. Harada, T. *et al.* Circularly polarized luminescence of Eu(III) complexes with point- and axis-chiral ligands dependent on coordination structures. *Inorg. Chem.* **48,** 11242–11250 (2009).
